# Swedish medical students' expectations of their future life

**DOI:** 10.5116/ijme.4ec5.92b8

**Published:** 2011-11-24

**Authors:** Saima Diderichsen, Jenny Andersson, Eva E. Johansson, Petra Verdonk, Antoine Lagro-Janssen, Katarina Hamberg

**Affiliations:** 1 Department of Public Health and Clinical Medicine, Family Medicine, Umeå University, Sweden; 2 VU University Medical Center, Dept. Medical Humanities, EMGO Institute for health and care research, the Netherlands; 3 Radboud University Nijmegen Medical Centre, Department of Primary and Community Care, Centre for Family Medicine, Geriatric and Public Health. Unit Women’s Studies Nijmegen, the Netherlands

**Keywords:** Medical students, gender, future expectations, physician’s role, work-life balance

## Abstract

Objectives: To investigate future life expectations among male and female medical students in their first and final year.

**Methods:**

The study was cross-sectional and conducted at a Swedish medical school. Out of 600 invited students, 507 (85%) answered an open-ended question about their future life, 298 (59%) first-year students and 209 (41%) last-year students. Women constituted 60% of the respondents. A mixed model design was applied; qualitative content analysis was utilized to create statistically comparable themes and categories.

**Results:**

Students’ written answers were coded, categorized and clustered into four themes: “Work”, “Family”, “Leisure” and “Quality of personal life”. Almost all students included aspects of work in their answers. Female students were more detailed than male ones in their family concerns. Almost a third of all students reflected on a future work-life balance, but considerations regarding quality of personal life and leisure were more common among last-year students.

**Conclusions:**

Today’s medical students expect more of life than work, especially those standing on the doorstep of working life. They intend to balance work not only with a family but also with leisure activities. Our results reflect work attitudes that challenge the health care system for more adaptive working conditions. We suggest that discussions about work-life balance should be included in medical curricula.

## Introduction

As in several other Western countries, Sweden has seen a marked increase of female medical students over the past century.^1^^-^^5^ In a decade or two, this changing composition in medical schools will replace the earlier male predominance with a slight majority of female physicians.^1^^,^^5^ A discussion of the possible consequences of this so-called “feminization” of medicine is in progress, and one concern that has been described is a future shortage of doctors.^5^ This concern is primarily based on women physicians working fewer hours than their male counterparts^5^^-^^9^ and the fact that they are underrepresented in some specialties, such as general surgery and orthopedics, while they tend to favor others, such as gynecology and dermatology.^5^^,^^9^^-^^11^ Parallel to this discussion on workforce implications of feminization, attention has been paid to a younger generation of physicians who might have other attitudes toward work than older colleagues. Worries have been raised that the younger generation of doctors are less committed and less hard-working.^12^ In order to understand and predict changes in attitudes towards work, it is not enough to look at the working patterns of today’s physicians. We need to ask our future doctors. This is important, as attitudes, values and aspirations have long-term impact on labor market behavior and outcomes.^13^ Research shows that medical students express concerns about how to combine a professional career with a life outside work.^14^^-^^16^ In the US, a so-called controllable lifestyle has become a determinant in students’ specialty selection criteria. In a Scandinavian study, Aasland et al.^14^ showed that when students chose their career after completion of medical school, the importance of having a balance between work and private life was important.The concept of work-life balance and how to define it has been an important topic in research.^17^ Clark^18^ described how balance is attained by managing and negotiating the work and family spheres and the borders between them. Work-life balance can be defined as “satisfaction and good functioning at work and at home, with a minimum of role conflict”.^18^ By naming the domain outside work home/family it is implied that this is mainly a women’s issue.More knowledge about students’ attitudes and concerns regarding their future career are important for health care planners and also for medical teachers involved in education. What are the crucial work-life issues for today’s students? Hitherto, most research about medical students’ preferences and ideas about their future has been conducted using specific questions about workload and specialty preferences or questionnaires with fixed answers to choose among. What do students wish for in the future if they can formulate the answer themselves?The aim of this cross-sectional study was to investigate the future life expectations among women and men at the beginning and at the end of medical school, by way of an open-ended question.

## Methods

### Study design

This study was cross-sectional and the data consisted of first- and last-year medical students’ answers to an open-ended question about their expectations of future life. It was the first question in a questionnaire about gender aspects in medicine. The students were asked to describe their ideal future: “Ideally when I graduate as a physician, my life will look as follows (in the next 10–15 years).” This question was derived from Hoffnung^19^, who used it to study college women’s expectations of career, marriage and motherhood.The open-ended question was followed by background questions about the participants’ age, sex and civil status, and specific questions concerning attitudes to gender issues in medical work and career (not analyzed in this paper).The participants were medical students at Umeå University in Sweden. Twice a year, every January and September, a new class enters the medical school. During a two-year period all first-year students from four classes (fall 2006 – spring 2008), and all last-year students from four classes (spring 2007– fall 2008) were invited. The first-yearstudents filled out the questionnaire in their first week of medical school and the last-year students at the end of their 11th and last semester. Participation was voluntary and those agreeing to participate stayed on after an ordinary lecture to answer the confidential questionnaire.The Ethics Committee at Umeå University has approved this study.

### Analysis

Data were analysed using a sequential mixed model design,^20^ meaning that initial inductive analysis of the free-text answers was conducted and followed by statistical analysis of the elaborated categories. In the first part qualitative content analysis was used^21^ to elaborate categories that could be analyzed quantitatively in the second part.

#### Part 1: Elaborating categories

To become familiar with the data and to obtain a sense of the whole, all answers were read by the first author (SD) in search of reoccurring content that could be clustered into categories. The impressions and ideas of this qualitative step were discussed in joint sessions with all authors.Three authors (SD, EEJ, KH) separately read and categorized the answers from 60 students according to content and meaning. By comparing the categorizations, the labeling of categories was discussed and reformulated. Based on this, another 60 answers were read, categorized and discussed. This procedure was repeated a few times until the categories created seemed consistent and enough varied to capture the diversity of the answers. A coding schedule of 34 categories was set up, clustered into four emergent themes: “Work”, “Family”, “Leisure” and “Quality of personal life”. The three authors who were involved in the categorizations had no access to information about the students such as sex and age.The first author (SD) performed the main encoding of all answers by filling out the schedule. If the category was represented in an answer it was marked with 1, otherwise with 0. One answer would normally be represented in several categories.In order to create a more lucid presentation of the results, the 34 categories were reduced to 18 by merging similar small categories. For example, the category “Hobbies” included less than 8% of the students and therefore was merged with “Spare-time travel” into the larger category “Travel and hobbies”.

#### Part 2: Quantitative analysis

Proportions of themes and categories were compared between men and women, first- and last-year students, using Pearson chi-square test (significance at p < .05; specified at p < .01 and p < .001). SPSS 17.0 for Mac OSX was used.To check the reliability of the coding, three researchers (SD, KH, EEJ) encoded 100 randomly chosen answers separately according to the 34 categories used in the main coding. The categorizations were then compared. A total of 46 discrepancies were identified. With 100 answers, 34 categories and three researchers, the percentage of discrepancies was less than 1%. Themes (clustered categories), categories, and examples of quotations within each category are presented in Table 1.

**Table 1:** Themes (bolded), categories, category descriptions and examples of statements included in each category

Theme / Category		Description		Example
Work				
I like my job		Described work as stimulating and nice or having nice colleagues.		“I have a nice job with nice colleagues”
I know what I want to be		One or two specialties were mentioned in the answer. Research and career aspirations were also included here.		“I want to work as a general practitioner”
The world is my workplace		Mentioned working in another country or abroad with humanitarian aid.		“I will work in any country in the world”
I am a good doctor		Expressed being a secure or good physician.		“I am good at what I do”, “I am beginning to take on the physician’s role”
Full-time or more		Described working full-time or that they intended to work a lot.		“I work a lot”
Part-time		Mentioned working less than full-time or that they intend to work “a little”.		“I work 75%”
Work – not specified		Expressed something work-related without being specific enough to be included in any of the work categories.		“I work as a doctor”
Family			
Children		Mentioned children or that they might have children.		“Three children”
Partner		Intimate relations of any kind.		“I have found a life partner”
Family – not specified		Included family without specifying it as partner and/or children.		“Family”
Leisure			
Travel and hobbies		Described traveling in their leisure time or having a hobby such as playing the piano.		“Plenty of vacation and trips” “Acknowledged cat breeder”
Friends and relatives		Included relatives and friends in their answer.		“Have time to see relatives and friends without having bad conscience”
Physical activity		Mentioned doing physical exercise such as a sport.		“I exercise a lot”, “Climbing, skiing and outdoor life”
Leisure – not specified		Included leisure without specifying it, as friends, a leisure activity or a hobby.		“I have a meaningful leisure time”
Quality of personal life			
Work-life balance		Manageable or flexible workload, balance between work and private life meaning leisure and/or family.		“I work full-time but I want to have time for leisure as well”, “I do not work too much”
Happiness		Described happiness of some sort, such as having a loving family, good relationships, harmony or a happy life in general.		“Life is still exciting!”, “Children who give me joy”, “I live in harmony”
Health		Included being healthy or having healthy children.		“Me and my family are all healthy and doing well”
Wealth	Described a wealthy lifestyle or a high salary.		“Good salary, a pool inside”

## Results

Out of 600 invited students, 507 (85%) answered the open-ended question, 305 (60%) women and 202 (40%) men. The response rate was 87% among the women and 81% among the men. First-year students accounted for 298 answers (59%) and last-year students for 209 answers (41%), giving an answer rate of 90% among first-year students and 78% among last-year students. The final-year students were on average 5 years older (Table 2). Therefore it is not surprising that about half of the first-year students compared with more two thirds of the final-year students had a partner. Also, male students in their final year were more likely to have children (19%) compared with their first-year peers (4%).The answers to the open-ended question varied in length from one word to several sentences, and were on average 25 words long. As outlined in the methods section, the content of the answers was sorted in 18 categories, which in turn were clustered into four themes. Comparing the occurrence of the themes among all students showed that “Work” (96%) and “Family” (77%) were included by a majority of the students, while “Leisure” (38%) and “Quality of personal life” (51%), were slightly less frequent. Below we compare the distribution of answers in the themes and categories between first- and last-year students, and between male and female students.

**Table 2:** Student characteristics (N = 507)

Variable	Men		P value	Women		P value
	First year n = 125	Last year n = 77		First year n = 173	Last year n = 132	
	Mean (SD)			Mean (SD)		
Age	23 (4)	28 (3)	< .001	22 (4)	27 (3)	< .001
	n (%)			n (%)		
Civil Status						
Single	63 (50)	25 (32)	< .05	96 (55)	37 (28)	< .001
Partner	62 (50)	52 (68)		77 (45)	95 (72)	
Children						
No	119 (96)	62 (81)	< .001	162 (94)	121 (92)	NS
Yes	5 (4)	15 (19)		10 (6)	11 (8)	

NS= Non-significant

### Comparing first- and last-year students

Students in their first and final year differed significantly in three out of four themes, and in a majority of the categories (Table 3). “Work” was the most common theme in all groups, but it was more frequent in first-year students’ answers than in last-year students’ answers (first-year 97%, last-year 93%, p < .05). On category level, first-year students had significantly higher proportions in “I like my job” and in “The world is my workplace”, implying that first-year students more often described job satisfaction and working abroad. Still, “I like my job” was frequent among the last-year students as well (43%), suggesting that stimulation, colleagues and workplace attitudes were important at the end of medical school as well. Working with humanitarian aid, a subcategory in “The world is my workplace” was included by 9% of the first-year students compared to 3% of the last-year students (not shown in table). The category “I know what I want to be”, i.e., having plans for specialty, research or making career advancements, was significantly more common among last-year students. Comments about part-time work were significantly more frequent in last-year students (first-year 4%, last-year 13%, p = 0.000). There was also a tendency that last-year students more often included notions about being a good and confident doctor in their answers, i.e., statements within the category “I’m a good doctor”, although this was not a significant difference.

**Table 3:** Themes (bolded) and categories in medical students’ future life expectations (N = 507)

	First year Total N = 298 n (%)		Last year Total N = 209 n (%)		P value	Men				P value	Women				P value
						First year N = 125 n (%)		Last year N = 77 n (%)			First year N = 173 n (%)		Last year N = 132 n (%)		
Work	290	(97)	195	(93)	< .05	122	(98)	65	(84)	< .001	168	(97)	130	(98)	NS
I like my job*	158	(53)	89	(43)	< .05	55	(44)	26	(34)	NS	103	(60)	63	(48)	< .05
I know what I want to be	70	(23)	82	(39)	< .001	28	(22)	26	(34)	NS	42	(24)	56	(42)	< .01
The world is my workplace	56	(19)	13	(6)	< .001	21	(17)	3	(4)	< .01	35	(20)	10	(8)	< .01
I am a good doctor	30	(10)	32	(15)	NS	12	(10)	8	(10)	NS	18	(10)	24	(18)	NS
Full-time or more	27	(9)	15	(7)	NS	9	(7)	3	(4)	NS	18	(10)	12	(9)	NS
Part-time	11	(4)	28	(13)	< .001	2	(2)	12	(16)	< .001	9	(5)	16	(12)	< .05
Work – not specified*	42	(14)	28	(13)	NS	25	(20)	11	(14)	NS	17	(10)	17	(13)	NS
Family	228	(77)	160	(77)	NS	89	(71)	53	(69)	NS	139	(80)	107	(81)	NS
Children*	136	(46)	119	(57)	< .05	51	(41)	35	(45)	NS	85	(49)	84	(64)	< .05
Partner	107	(36)	100	(48)	< .01	49	(39)	33	(43)	NS	58	(34)	67	(51)	< .01
Family – not specified	72	(24)	28	(13)	< .01	27	(22)	10	(13)	NS	45	(26)	18	(14)	< .01
Leisure	93	(31)	98	(47)	< .001	36	(29)	33	(43)	< .05	57	(33)	65	(49)	< .01
Travel and hobbies*	37	(12)	50	(24)	< .01	14	(11)	11	(14)	NS	23	(13)	39	(30)	< .001
Friends and relatives	35	(12)	42	(20)	< .05	8	(6)	18	(23)	< .001	27	(16)	24	(18)	NS
Physical activity	20	(7)	26	(12)	< .05	6	(8)	9	(12)	NS	12	(7)	17	(13)	NS
Leisure – not specified	17	(6)	16	(8)	NS	9	(7)	4	(5)	NS	8	(5)	12	(9)	NS
Quality of personal life	131	(44)	127	(61)	< .001	48	(38)	51	(66)	< .001	83	(48)	76	(58)	NS
Work-life balance	81	(27)	67	(32)	NS	24	(19)	26	(34)	< .05	57	(33)	41	(31)	NS
Happiness	31	(10)	50	(24)	< .001	13	(10)	21	(27)	< .01	18	(10)	29	(22)	< .01
Health	15	(5)	20	(10)	< .05	3	(2)	9	(12)	< .01	12	(7)	11	(8)	NS
Wealth	39	(13)	39	(19)	NS	20	(16)	15	(19)	NS	19	(11)	24	(18)	NS

NS= Non-significant; * there were significant differences between male and female medical students (p<0.05).

The proportion of answers containing aspects of the theme “Family” was similar in first- and last-year students. However, the proportions of the categories in “Family” varied between the groups. Last-year students were more specific and significantly more often included partner and children, compared with first-year students who more often just mentioned family without going into any details.The theme “Leisure” was more frequent in the answers from last-year students (first-year 31%, last-year 47%, p = 0.000). Compared with first-year students they had significantly higher proportions in the categories “Friends and relatives”, “Travel and hobbies” and “Physical activity”.The same pattern as in “Leisure” was seen in “Quality of personal life”. While 61% of the last-year students’ answers were represented in this theme, the corresponding proportion among first-year students was 44% (p = 0,000). On category level it was significantly more common among last-year students to write about “Happiness” and “Health”. The category “Wealth”, including for example descriptions of a large house, an expensive car or a high salary, occurred in 13% of the first-year students compared with 19% of the last-year students.

### Comparing male and female first- and last-year students

Table 3 shows that the differences described above between first- and last-year students were related to gender. In the theme “Work”, the difference between first- and last-year students study was explained by the fact that male – not female – last-year students mentioned work aspects less often than their first-year counterparts. The higher proportions of the categories “I know what I want to be” and “I am a good doctor” in last-year students’ answers was chiefly explained by the fact that last-year women emphasized those aspects more than other participants did. A relatively small proportion of the students mentioned working “part-time”, but interestingly the largest difference was displayed among the male students (first-year 2%, last-year 16%).Last-year students being more specific about family aspects were a result of the female students’ considerations. Among women there was a shift from “Family – not specified” in their first year to “Children” and “Partner” in their final year. For example, they described how many children they planned for or gave details about their partner. The higher proportions of last-year students represented in the theme of “Leisure” were explained by the fact that women in their final year emphasized “Travel and hobbies” more often than other students and that male last-year students were more concerned with “Friends and relatives” than their first-year peers.

The higher frequency of last-year students included in the theme “Quality of personal life” can chiefly be explained by the large difference between men in their first and final year. The same pattern was seen in the category “Work-life balance”, which occurred in similar proportions in first- and last-year women’s answers, whereas male last-year students included “Work-life balance” significantly more often (34%) than first-year ones (19%). Looking more closely at the answers in “Work-life balance”, it was noticed that 53% of the female answers specified “Work-life balance” as having time for family, while the corresponding proportion among male students was 36% (not shown in table).

When comparing the answers between all men and all women, we found some significant differences (marked with a superscript “a” in table 3). More women wrote about work and family aspects and their answers were more often included in the categories, “I like my job”, “Children” and “Travel and Hobbies”. The male students’ answers were more often than their female peers sorted in the category “Work – not specified”.

## Discussion

This cross-sectional study explored future life expectations of medical students by means of an open-ended question. The analysis of the free-text answers revealed several differences between first- and last-year students as well as gender differences.Almost all students mentioned work, but the work theme was less frequent among men in their last year. Male last-year students also mentioned “Quality of personal life” and “Leisure” more often than their first-year peers.

The female students in their final year were both more work-oriented and went into more detail about family than their male peers. Furthermore, nearly half of the female last-year students described leisure activities as part of their ideal future. “Work-life balance” was considered by over 30% of both male and female last-year students. All in all, most students were work-oriented, but they also made it clear that they want more out of life than work.The medical students in our study seemed unwilling to sacrifice private life for work, as has been observed before in both medical students and young physicians.^12^^, ^^14^^-^^16^^, ^^22^^-^^24^ Emslie and Hunt^25^ described two groups of male employees; one group who “live to work” and another group who “work to live”, emphasizing the importance of life outside work. Perhaps today’s male and female medical students intend to “work to live” rather than “live to work”. This trend towards a more controllable lifestyle could be a reaction against the heavy workload of today’s physicians. In Sweden, 55% of the physicians report that they are either too tired or have too little time for private life.^26^ Such conditions among the students’ clinical role models might contribute to the desire for work-life balance and the wish for a richer life. This would also explain why last-year students with more clinical experience were more focused on family, leisure and quality of personal life than first-year ones. It should however be added that last-year students – being on average 5 years older – were closer to a phase of family planning.Hakim^13^ described three sociological ideal types based on lifestyle preferences of women’s labor market careers: home-centered, adaptive and work-centered. The women in our study seemed work-centered and adaptive at the same time, wanting time for it all: work, family and leisure. Their future expectations are not characterized as “either/or”, but rather as “work and more”. Adding to earlier research, we found that these work attitudes are expressed among Swedish male students as well, suggesting that the desire for a manageable workload and a rich life outside work is more gender neutral than expected. Still, part-time work is significantly more common among women physicians, even if part-time work is increasing among men as well. ^6^^-^^8^ In Sweden, the proportion of women physicians practicing part-time increased from 26% in 2000 to 29% in 2009, which can be juxtaposed with the proportion of male part-timers increasing from 11% in 2000 to 17% in 2009.^6^^,^^7^ Thus, our findings indicate that this trend where part-time becomes more gender neutral, is only the very beginning of a future where men will demand a manageable workload to a similar extent as women. This means that the predicted shortage of doctors should not be discussed in terms of a feminization of medicine^5^ but rather in terms of priorities in the generation of today.A previous Swedish study showed male medical students expecting a future with time for hobbies, whereas female students were concerned with how to combine work with family.^15^ This gendered pattern can be seen in our study as well; women students plan for family to a larger extent, which reflects the working patterns of today where mainly female physicians consider family responsibilities when planning their career.^11^ Thus, even though part-time work is slightly more gender-neutral today,^6^^,^^7^ the impact of family responsibilities when choosing specialty and part-time is still divided along traditional gender lines.^11^^,^^14^^, ^^27^When discussing work-life balance Clark^18^ and Hakim^13^ outline two domains: home/family and work. However, our results suggest that there is a third important domain: leisure, where personal spare time is spent. When work and family involve satisfying other people’s needs, physical activity, spending time with friends and hobbies can fulfill personal needs. Thus, medical students are work-centered but intend to balance and negotiate the work domain not only with a home domain but also with a leisure domain. Perhaps it is when these three domains are balanced that “Quality of personal life” is achieved.

### 

#### Strengths and weaknesses

This study used an open-ended question in a questionnaire to explore medical students’ future life expectations. To prevent the free-text answer from being biased by the other questions, we put the open-ended question first. Drawing on free-text answers as data can be considered a strength, as it gave the opportunity to grasp what was in the students’ minds, their immediate and condensed thoughts.We applied an innovative approach using a mixed model design^20^ where statistical analysis was performed on qualitatively evolved categories. Basing an analysis on short answers to an open-ended question might limit the depth of the result. On the other hand, a considerable number of participants were included, and although some answers were only a few words, the total material was rich and generated a large number of categories. The systematic construction of categories that were grounded in the data and the reliability test of the coding were done to achieve trustworthy results. The inter-rater reliability test showed a high consensus, suggesting that the categories were clear and consistent.A mean response rate of 85% is high, though it varied between first-year (90%) and last-year students (78%). Last-year students being on clinical training out of town can in part explain the lower response rate among them. However, since the proportion of male final-year students was higher among non-responders there might be a selection bias in responders and non-responders. This is a cross-sectional study and not a cohort study following the same students through medical school. Consequently the groups may intrinsically differ from each other. However, since four separate first- and last-year courses were invited, we estimate this risk to be minor. The first- and last-year students were in different phases of life in the sense that students at their final year were on average 5 years older and a vast majority of them lived with their partner, whereas the first-year students had just entered medical school and more than half of them were single. These differences might explain the first-year students being less specific about family and should be considered in the overall comparisons between first- and last-year students.The results are derived from a single medical school, which means that our findings are not necessarily representative of medical students in general. We must also keep in mind that the students’ comments represent personal considerations as well as social discourses related to the study being conducted at a Swedish university.

## Conclusions

The medical students of today want more to life than work. The doctor’s vocation is known for its long hours and heavy workload, yet when asked about their ideal future in their first week of medical school more than a fourth reflect on how to obtain a work-life balance. Not only a family domain was included in this balance; the medical students in our study wanted time for leisure activities as well. This was accentuated at the end of medical school, suggesting that future physicians will emphasize the importance of family, leisure and quality of personal life, but not at the expense of their work. They want it all. Moreover, the trend – more to life than work – was more pronounced among men. This is interesting as a manageable workload is often juxtaposed with women’s desire to have more time for family. Even though the female students were more family-oriented, our study shows that they were just as work-oriented as their male peers. Feminization as the reason for increased part-time work is thereby challenged by gender-neutral desires for work-life balance. These work attitudes and values among future physicians are a challenge for health care workforce planning and call for more adaptive working conditions in all specialties. The question is whether the health care system and the old-time colleagues are ready, not only for more male and female physicians demanding part-time to take care of family duties, but also for those who justify part-time work by having more time for leisure activities.Part-time work might be one way to achieve work-life balance. However, we suggest that it is important for policy makers and health care planners to plan for full-time jobs that enable time for family and leisure activities as well. For medical educators this study shows that male as well as female students have concerns about how to combine work and private life in the future, and we therefore suggest that discussions about work-life balance should be include in the medical curricula.

### Conflict of Interest

The authors declare that they have no conflict of interest.
